# Effects of intradialytic exercise on frailty in maintenance hemodialysis patients: a systematic review and meta-analysis 

**DOI:** 10.3389/fphys.2025.1600219

**Published:** 2025-11-06

**Authors:** Zhao Hua Zou, Ji Quan Zhang, Zi Han Yi, Xing Chen, Wei Qing

**Affiliations:** 1 Nephrology Department, Deyang People’s Hospital, Deyang, China; 2 Department of Nursing, Aobaoka Hospital, Osakasayama, Japan

**Keywords:** intradialytic exercise, hemodialysis, frailty, effects, meta-analysis

## Abstract

**Background:**

The pooled prevalence of frailty in maintenance hemodialysis patients is increasing, and research on the effects of intradialytic exercise to improve frailty remains limited.

**Objectives:**

To analyze the effects of intradialytic exercise on frailty in maintenance hemodialysis patients through randomized clinical trials and quasi-experimental studies.

**Methods:**

We performed a comprehensive literature search in PubMed, Embase, Web of Science, and Cochrane Library, and English-language publications were indexed from January 2010 to August 2024. Statistical analyses were performed using Review Manager V.5.3 and STATA 15.0. Statistical heterogeneity among studies was quantified using the Chi-square and I-square tests, and publication bias was evaluated using Egger’s test and funnel plots.

**Results:**

31 studies involving 1,365 maintenance hemodialysis patients were included. The data from the meta-analysis showed that intradialytic exercise significantly reduced frailty score (MD = −0.98, 95%CI: 1.90 to −0.06, *p* = 0.04) and fatigue (SMD = −0.47, 95%CI: 0.72 to −0.23, *p* = 0.0001). Also, intradialytic exercise significantly increased grip strength (MD = 2.42, 95%CI:0.78 to 4.06, *p* = 0.004), 6-min walking distance (MD = 36.65, 95%CI:24.90 to 48.39, *p* < 0.0001), and step counts (SMD = 0.32, 95%CI:0.04 to 0.60, *p* = 0.03). However, no significant effects were found in body weight (MD = 0.71, 95%CI: 1.28 to 2.69, *p* = 0.48).

**Conclusion:**

Intradialytic exercise can significantly improve overall frailty and frailty indicators such as grip strength, 6-min walking distance, step counts, and fatigue. Thus, intradialytic exercises might be a viable strategy for frailty in maintenance hemodialysis patients.

**Systematic Review registration:**

CRD42024576582.

## 1 Introduction

Frailty is a nonspecific clinical state characterized by decreased physiological reserves, increased vulnerability, and diminished stress resistance (Fried et al., 2001), accompanied by an increase in adverse events, including falls, delirium, disability, hospitalization, and death (Buckinx et al., 2015; Kojima et al., 2019). The assessment of overall frailty primarily employs standardized scales. The most commonly used frailty assessment tool is Fried’s Frailty Phenotype (Fried et al., 2001), which includes five indicators of weakness (grip strength), slow walking speed, low physical activity, self-reported exhaustion, and unintentional weight loss. It is considered frail if at least three of these five indicators are present. Frailty is highly prevalent in patients with chronic diseases and has become a hot topic among kidney disease researchers in recent years.

Chronic kidney disease (CKD) is a clinical syndrome of progressive and irreversible decline in kidney function. The global prevalence of CKD is 9.1% (GBD Chronic Kidney Disease Collaboration, 2020). Maintenance hemodialysis (MHD) is the primary renal replacement therapy for treating end-stage renal disease (ESRD) patients. MHD patients undergoing long-term hemodialysis are more prone to frailty due to continuous loss of nutrients, decreased muscle strength, and low physical activity. The pooled prevalence of frailty in MHD patients is 46% (Lee and Son et al., 2021), which is 3–10 times higher than in community-dwelling older adults (Nitta et al., 2018). Frailty is an independent predictor of hospitalization and death in MHD patients (McAdams-DeMarco et al., 2013). However, the frailty state is dynamically reversible, and if it can be recognized and intervened promptly, the condition can be delayed or even reversed, and the prognosis can be improved.

The International Conference of Frailty and Sarcopenia Research (ICFSR) published clinical practice guidelines. It recommended that first-line therapy for managing frailty should include a multi-component physical activity program (Dent et al., 2019). Physical activity is the most feasible way to prevent and treat frailty, and it may be ideally suited for ESRD patients on dialysis (Harhay et al., 2020). However, there is insufficient evidence to determine the optimal frequency, intensity, duration, and type of physical activity necessary to manage frailty and the optimal combination of aerobic and resistance exercise.

Five systematic reviews summarized the effects of exercise interventions on MHD patients (Yoo et al., 2022; Li et al., 2024; Neto et al., 2018; Young et al., 2018; Wahida and Rumahorbo, 2022; Yoo et al., 2022 reported 13 studies of home-based exercise that included aerobic exercise, resistance exercise, and behavioral components that significantly improved strength, walking speed, and physical activity, and home-based exercise may be effective in improving specific frailty indicators. However, home-based exercise for dialysis patients has low exercise compliance and lacks adequate supervision. Li et al., 2024 reported 9 studies that showed that intradialytic exercise significantly improved grip strength and 6-min walking distance (6MWD). Neto et al., 2018 reported that 24 meta-analysis studies showed that intradialytic exercise significantly improved 6MWD. Young et al., 2018 reported 8 studies that showed that intradialytic exercise significantly improved 6MWD. Wahida and Rumahorbo, 2022 reported that 15 studies showed that intradialytic exercise significantly improved fatigue. The above four studies of intradialytic exercise included aerobic exercise, resistance exercise, and combined exercise, which could improve the physical function of MHD patients. However, no studies of frailty score were reported, nor were studies of all five frailty indicators reported.

Because the evidence for the effect of intradialytic exercise on frailty in MHD patients remains uncertain, this study conducted a systematic review and meta-analysis to provide strong evidence that intradialytic exercise improves frailty and to summarize prescriptions for intradialytic exercise to better guide clinical practice.

## 2 Methods

### 2.1 Design

This study was completed using the Preferred Reporting Items for Systematic Reviews and Meta-Analyses (PRISMA) guidelines (Page et al., 2021). It has been registered in the International Prospective Register of Systematic Review (PROSPERO): CRD42024576582.

### 2.2 Eligibility criteria

According to PICOS ((Population, Intervention, Comparison, Outcomes, and Study design) principles (Liberati et al., 2009), we determine the following inclusion criteria: (1) The population were adult patients on MHD for at least 3 months; (2) Intervention group receiving intradialytic exercise (including aerobic exercise or/and resistance exercise); (3) Control group receiving usual care (no exercise or simple stretch); (4) The outcome measures were frailty score or represented at least one frailty indicator (e.g., grip strength, walking speed, physical activity, fatigue, and body weight); (5) randomized clinical trials (RCTs) and quasi-experimental studies. exclusion criteria: (1) without available full text; (2) review; (3) case report; (4) conference or letter; (5) not published in English Language.

### 2.3 Search strategy

We searched the literature in four databases, including PubMed, Embase, Web of Science, and Cochrane Library; all the English-language publications were indexed from January 2010 to August 2024. We also reviewed the reference lists of the cited literature to identify other relevant studies. Combinations of controlled vocabulary using Medical Subject Heading (MeSH) terms and free-text terms. The search terms included renal dialysis, hemodialysis, hemodialyses, haemodialysis, hemodiafiltration, haemodiafiltration, exercise, training, intradialytic exercise, intradialytic training, aerobic exercise, aerobic training, resistance exercise, resistance training, frailty, frailties, frailness, debility, debilities, hand strength, grip strength, walking speed, gait speed, walking pace, activities of daily living, daily living activity, physical activity, fatigue, exhaustion, lassitude, body weight, body mass. Boolean operators “OR, AND” were used for the search (Supplementary File 1).

### 2.4 Study selection and data extraction

Two researchers independently performed the literature search, screening titles and abstracts, full-text reviews, study quality assessment, and data extraction. Any disagreement between two researchers was resolved by discussion until a consensus was reached or by consulting a third researcher. The extracted data included the first author, publication year, country, sample size, exercise modality, exercise frequency, trial duration, exercise protocol, and outcomes.

### 2.5 Quality assessment of the study

Two researchers used version 2 of the Cochrane Risk of Bias tool for RCTs (ROB 2) and Joanna Briggs Institute (JBI) adapted quasi-experimental study evaluation tool to assess the quality of the included studies (Sterne et al., 2019; Barker et al., 2024). The ROB 2 included the randomization process; deviation from the intended intervention; missing outcome data; outcome measures; selection of the reported result; and overall, each indicator contains three levels: low-risk, unclear, and high-risk. The JBI adapted quasi-experimental study evaluation tool included temporal procedure bias; selection and allocation bias; confounding factors bias; administration of intervention/exposure bias; outcome assessment, detection and measurement bias; and participant retention bias, each question can be scored as yes, no, unclear, or not applicable. Any disagreement was resolved by consensus or third-party adjudication.

### 2.6 Data synthesis and analysis

The treatment effect was measured as the mean and standard deviation (SD) change between the pre-and post-intervention measurements. Continuous data were pooled as the mean differences (MD) and 95% confidence intervals (95%CI) if the outcome measuring methods and units were identical among studies; otherwise, the standardized mean differences (SMD) were used. Statistical heterogeneity among studies was quantified using the Chi-square and I-square (I^2^) tests. If *p* < 0.10 or I^2^>50%, there was significant heterogeneity among studies; the random-effects model was used to combine the data. Otherwise, a fixed-effects model was used. We used the visual assessment of funnel plots and Egger’s test to evaluate publication bias (Egger et al., 1997). We used sensitivity analysis to investigate the robustness of the meta-analysis models. Statistical analyses were performed using Review Manager V.5.3 and STATA 15.0.

## 3 Results

### 3.1 Search outcome

**FIGURE 1:**
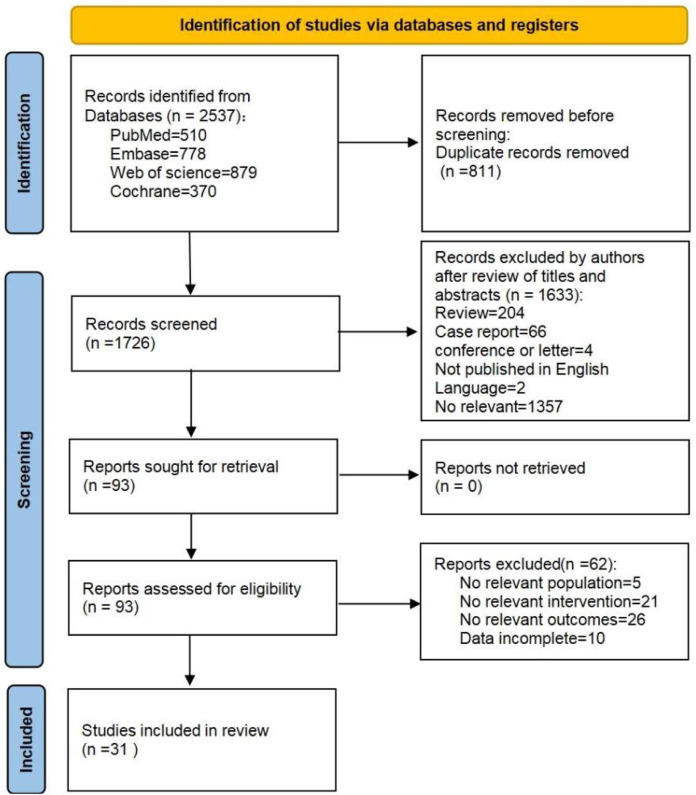
PRISMA flow diagram regarding article selection for meta-analysis.

A total of 2,537 studies were identified from four databases: PubMed, Embase, Web of Science, and the Cochrane Library, and 811 duplicate studies were removed using NoteExpress 3.5 software. After screening the titles and abstracts, 93 studies were selected for full-text screening, and 62 studies were excluded according to the inclusion criteria. Finally, 31 studies were included in the systematic review and meta-analysis. The flow diagram of search and selection is shown in Figure 1.

### 3.2 Study characteristics

A total of 31 studies involving 1365 MHD patients were included in this study. Among these, 27 studies were RCTs, and 4 studies were quasi-experimental studies. 11 studies were conducted in Europe, 6 studies were conducted in South America, 13 studies were conducted in Asia, and 1 studies were conducted in Oceania. All patients had been receiving hemodialysis for at least 3 months; 669 patients were in the intervention group receiving intradialytic exercise (aerobic exercise or/and resistance exercise), and 696 patients were in the control group receiving usual care. 17 studies included only aerobic exercise by cycle ergometer, 8 studies included resistance exercise by elastic bands, elastic balls, dumbbells, or body weight, and 6 studies included aerobic exercise and resistance exercise. The intervention details among the studies are as follows: the exercise duration ranged from 6 to 48 weeks, the exercise frequency was either 2 or 3 times per week, most of the exercise time was 30–60 min, and most of the exercise was completed between the first and second hours of the hemodialysis session. The exercise intensity is appropriate for low to moderate-intensity exercise at 60–70% of maximal heart rate or 12-16 on the Borg Perceived Exertion Scale. The basic characteristics of the included studies are shown in Table 1.

**TABLE 1:** Basic characteristics of the included studies.

First author, year	Country	Sample,n (I/C)	Trial duration	Frequency	Exercise type	Exercise protocol	Outcomes
Kim et al. (2022)	Korea	18/21	3 months	3 times/week	AE	The exercise included a 5-min warm-up,30–60 min of cycling, and a 5-min cool-down, at an intensity of 12–15 on the Borg Perceived Exertion Scale	frailty score, grip strength
Desai et al. (2019)	United Kingdom	13/21	4 months	3 times/week	AE	Each exercise session consisted of three phases: warm-up, cycling and cool-down, the patients were asked to cycle optimally aiming for their Rated Perceived Exertion between 13 and 15	frailty score, grip strength, body weight
Sánchez-Tocino et al. (2022)	Spain	18/33	3 months	3 times/week	AE and RE	Patients performed aerobic and resistance exercises for 60 min at an intensity of 12–14 on the Borg Perceived Exertion Scale, using elastic bands, weighted ankle braces, foam balls, pilates rings and foot peddler	frailty score
Koh et al. (2010)	Australia	15/14	6 months	3 times/week	AE	Cycling for 15–45 min at an intensity of 12–13 on the Borg Perceived Exertion Scale	grip strength, 6MWD
Wu et al. (2014)	China	32/33	3 months	3 times/week	AE	Patients performed a 5-min warm-up and 10–15 min of cycling, at an intensity of 12–16 on the Borg Perceived Exertion Scale	grip strength, 6MWD
Krase et al. (2022)	Greece	21/23	7 months	3 times/week	AE	Cycling for 60 min at an intensity of 60% of the patient’s maximal exercise capacity	grip strength, 6MWD, step count
Sovatzidis et al. (2020)	Greece	10/10	6 months	3 times/week	AE	Each exercise included a 5-min warm-up, cycling at the desired workload for a self-selected time, and a 5-min cool-down, at an intensity of 11–13 on the Borg Perceived Exertion Scale	grip strength, body weight
Dong et al. (2019)	China	21/20	3 months	3 times/week	RE	Patients performed a 5-min warm-up followed by a 1–2 h resistance exercise with high or moderate intensity, using their body weight and elastic balls	grip strength
Zhang et al. (2020)	China	43/44	3 months	2 or 3 times/week	RE	The resistance exercise included a 5-min warm-up,30–40 min of exercise applied to the wrist and ankle, and a 5-min cool-down, at an intensity of 12–13 on the Borg Perceived Exertion Scale	grip strength, 6MWD
Lopes et al. (2019)	Brazil	16/20	3 months	3 times/week	RE	Patients performed resistance exercises for 20–40 min until volitional fatigue occurred, using their ankle weights and elastic bands	grip strength, body weight
Cardoso et al. (2020)	Brazil	20/18	3 months	3 times/week	AE	Cycling for 20 min at an intensity of 60%–76% of maximal heart rate	6MWD
Dobsak et al. (2012)	Czech Republic	11/10	5 months	3 times/week	AE	Each exercise included a 5-min warm-up, 20–40 min of bicycle training, and a 5-min cool-down	6MWD
Vogiatzaki et al. (2022)	Greece	12/12	6 months	3 times/week	AE	The exercise included a 5-min warm-up, 30–50 min of cycling, and a 5-min cool-down, at an intensity of 13–14 on the Borg Perceived Exertion Scale	6MWD
Fernandes et al. (2019)	Brazil	20/19	2 months	3 times/week	AE	The exercise included a 10-min warm-up, 30 min of cycling, and a 10-min cool-down, at an intensity of 50%–70% of maximal heart rate	6MWD
Liu et al. (2023)	China	42/42	12 months	3 times/week	AE	The aerobic exercise included an ascending period of 5 min, a flat period of 15 min, and a descending period of 5 min, the exercise intensity was maintained at 5 to 15 points on the cardiopulmonary exercise test report	6MWD
Groussard et al. (2015)	France	8/10	3 months	3 times/week	AE	The exercise included a 5-min warm-up,15–30 min of cycling, and a 5-min cool-down, at an intensity of 55%–60% of the patient’s maximal exercise capacity	6MWD, body weight
Valle et al. (2020)	Brazil	12/12	3 months	3 times/week	RE	Two to three sets of 10 repetitions of resistance exercise were performed with weighted ankle cuffs and dumbbells, maintaining the Borg scale rating between 3 and 5	6MWD
Kirkman et al. (2014)	United Kingdom	9/10	3 months	3 times/week	RE	Patients completed three sets of 8–10 repetitions using a series of resistance bands, when 10-12 repetitions could be completed at a rating of perceived exertion below 15, the training load increased accordingly	6MWD
Exel et al. (2021)	Brazil	54/53	2 months	3 times/week	RE	Three sets of 10 repetitions of resistance exercise lasted 30 min with shin pads, initially with a 50% strength workload	6MWD
Yeh et al. (2020)	Taiwan	30/32	3 months	3 times/week	AE and RE	The exercise involved aerobic and resistance modalities by stationary cycling equipment, including a 5-min warm-up,20 min of main exercise, and a 5-min cool-down, the intensity was maintained at the Borg Perceived Exertion Scale of 12–14	6MWD
Marchesan et al. (2014)	Brazil	11/11	4 months	3 times/week	AE and RE	Warm up was performed for 3 min and the patient was submitted to aerobic exercises for 20 min, the load and volume of the localized muscle strength exercises were increased gradually, and the intensity aimed at 60%–70% of maximal heart rate	6MWD
Jamshidpour et al. (2020)	Iran	15/13	2 months	3 times/week	AE and RE	The exercise included a 3–5-min warm-up,20–45 min of static cycling and lower extremity resistance exercise training, and a cool-down, the intensity was maintained at the Borg Perceived Exertion Scale of 11–15	6MWD
Young et al. (2020)	United Kingdom	10/5	6 months	3 times/week	AE	Participants in the exercise group undertook cycling at a rating of perceived exertion of 12–14	step count
Brown et al. (2021)	United Kingdom	51/50	6 months	3 times/week	AE	Cycling for 30 min at an intensity of 12–14 on the Borg Perceived Exertion Scale	step count, body weight
Assawasaksakul et al. (2021)	Thailand	6/6	6 months	3 times/week	AE and RE	The exercise included a 10-min warm-up,10–30 min of aerobic exercise, 10 min of resistance exercise, and a 10-min cool-down, the intensity was maintained at the Borg Perceived Exertion Scale of 13	step count
Mihaescu et al. (2013)	Romania	18/14	3 months	3 times/week	RE	Two to three sets of 10 repetitions of resistance exercise were performed for 40 min with elastic bands, dumbbells and ankle weights, at an intensity of 12–14 on the Borg Perceived Exertion Scale	step count, body weight
Salehi et al. (2020)	Iran	20/17	3 months	2 times/week	AE	Patients performed passive pedaling at low power for 20 min during the first 2 h of every hemodialysis session	MFI
Malini et al. (2022)	Indonesia	23/24	2 months	2 times/week	AE	The exercise included a 5-min warm-up,20 min of pedaling movements, and a 5-min cool-down, at an intensity of 50%–60% of resting heart rate	FAS
Chang et al. (2010)	Taiwan	18/24	2 months	2 or 3 times/week	AE	The exercise included a 5-min warm-up and 10–30 min of cycling, at an intensity of 12–13 on the Borg Perceived Exertion Scale	HPFS
Huang et al. (2021)	Taiwan	40/43	3 months	3 times/week	RE	The low-intensity leg exercise comprised leg lifts, quadriceps femoris contraction, and knee flexion, and each section was repeated and lasted for 15 min	HPFS
Devagourou et al. (2021)	India	32/32	6 weeks	3 times/week	AE and RE	Six low-intensity intradialytic exercises were chosen (2 uppers and 4 lower limbs) which were repeated 10 times each over 1 min, the set of exercises were repeated thrice at an interval of 10 min	FACIT-F

Abbreviations: I, intervention group; C, control group; AE, aerobic exercise; RE, resistance exercise; 6MWD, 6 min walk distance; MFI, multidimensional fatigue inventory; FAS, fatigue assessment scale; HPFS, hemodialysis patients fatigue scale; FACIT-F, Functional assessment of chronic illness therapy-fatigue.

### 3.3 Risk of bias

**FIGURE 2:**
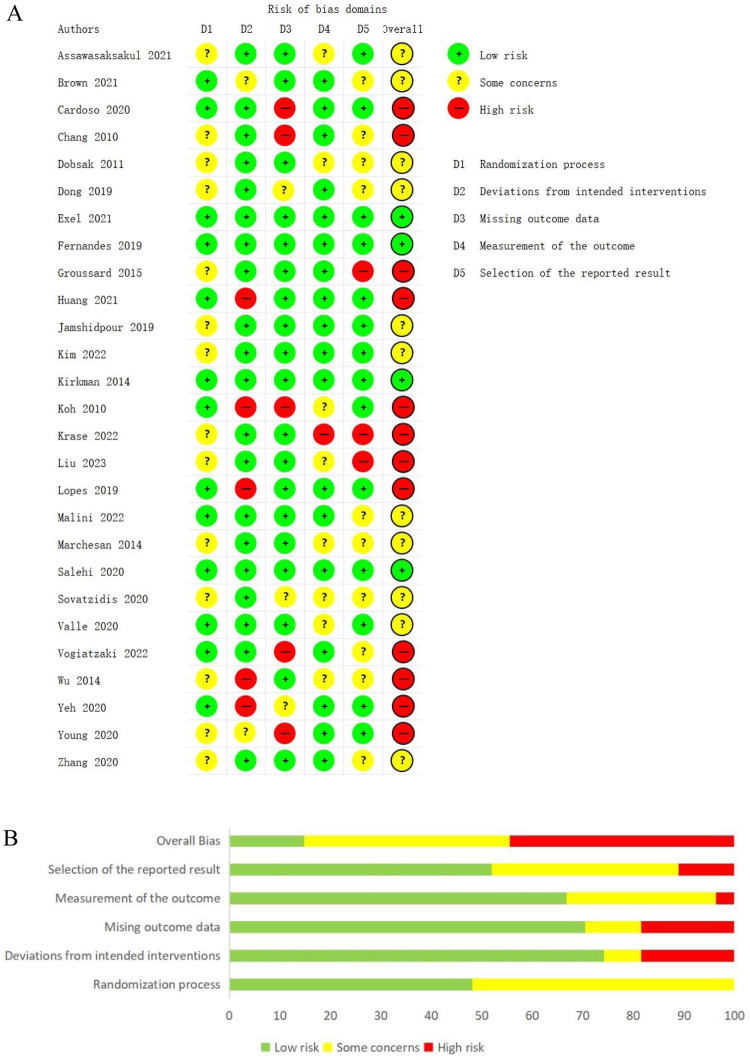
**(A)** Assessments of risk of bias for the included studies. **(B)** A summary of the risk of bias assessments for the included studies.

We used the ROB 2 tool to assess the risk of bias for 27 studies. 4 studies were considered to have a low risk of bias, 11 studies were at some risk of bias, and 12 studies were at a high risk of bias. Of these, 20 studies reported detailed randomization methods, and 13 described the allocation concealment process. Due to the nature of the intervention, we found that only 3 studies used the blinding method of participants or intervenors, 12 studies described the blinding of outcome assessors, and 15 studies had trial registration protocols. The results of the risk of bias are detailed in Figures 2A,B. We also used the JBI adapted quasi-experimental study evaluation tool to assess the risk of bias for 4 studies, of these, in the domain of ''participant retention bias'', Mihaescu et al., 2013; Sánchez-Tocino et al., 2022 were answered as no, other risks of bias domains were answered as yes. All risk of bias domains in Desai et al., 2019; Devagourou et al., 2021 were answered as yes.

### 3.4 Frailty indicators and effect sizes

#### 3.4.1 Frailty score

**FIGURE 3:**
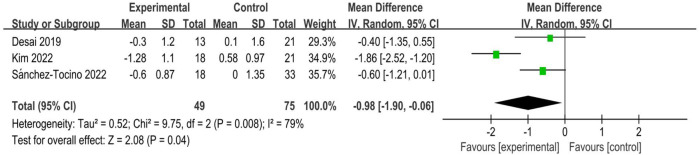
Forest plots of outcomes in frailty score.

Only 3 studies (Desai et al., 2019; Kim et al., 2022; Sánchez-Tocino et al., 2022) reported frailty scores using Fried’s Frailty Phenotype, involving 124 participants (49 in the experimental groups and 75 in the control groups). Statistical heterogeneity existed between the studies (I^2^ = 79%, p = 0.008), and after careful checking of the literature, heterogeneity could not be excluded, so the random effects model was chosen. The results showed that intradialytic exercise could reduce frailty score (MD = −0.98, 95%CI: 1.90 to −0.06, *p* = 0.04; Figure 3). Egger’s test was non-significant (t = 0.34, *p* = 0.792).

#### 3.4.2 Grip strength

**FIGURE 4:**
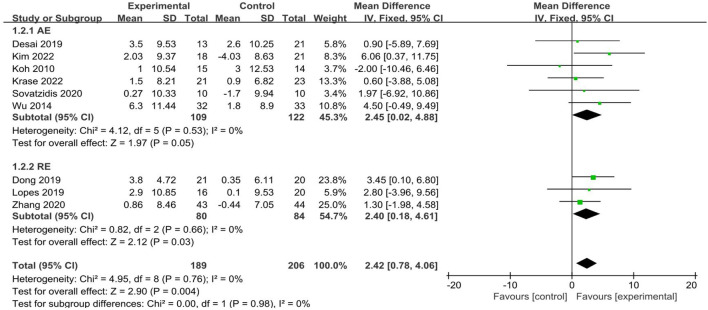
Forest plots of outcomes in grip strength.

A total of 9 studies (Desai et al., 2019; Dong et al., 2019; Kim et al., 2022; Koh et al., 2010; Krase et al., 2022; Lopes et al., 2019; Sovatzidis et al., 2020; Wu et al., 2014; Zhang et al., 2020) reported grip strength, involving 395 participants (189 in the experimental groups and 206 in the control groups). The analysis of data in the fixed effect model showed that intradialytic exercise could increase grip strength (MD = 2.42, 95%CI:0.78 to 4.06, *p* = 0.004; Figure 4), with a low level of heterogeneity (I^2^ = 0%, *p* = 0.76). Egger’s test was non-significant (t = −0.30, *p* = 0.773). We also used subgroup analyses to assess the effects of different exercise types and showed that aerobic exercise and resistance exercise both increased grip strength.

#### 3.4.3 Walking speed

**FIGURE 5:**
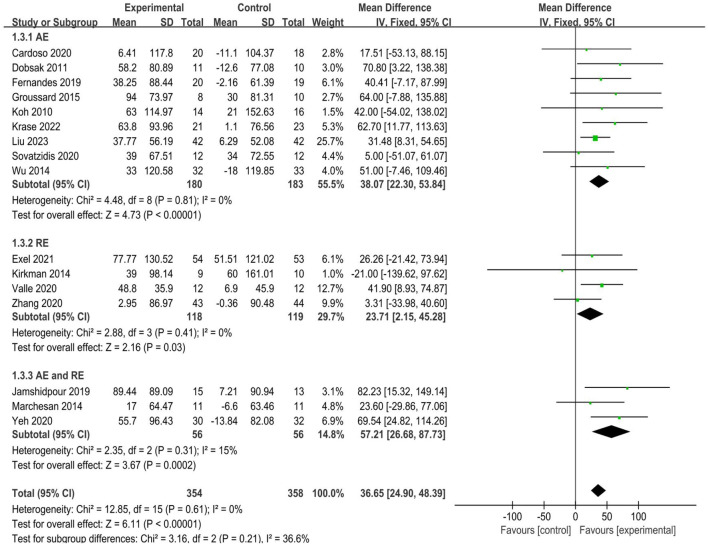
Forest plots of outcomes in 6MWD.

A total of 16 studies (Cardoso et al., 2020; Dobsak et al., 2012; Exel et al., 2021; Fernandes et al., 2019; Groussard et al., 2015; Jamshidpour et al., 2020; Kirkman et al., 2014; Koh et al., 2010; Krase et al., 2022; Liu et al., 2023; Marchesan et al., 2014; Valle et al., 2020; Vogiatzaki et al., 2022; Wu et al., 2014; Yeh et al., 2020; Zhang et al., 2020) reported walking speed, as determined by 6MWD, involving 712 participants (354 in the experimental groups and 358 in the control groups). The analysis of data in the fixed effect model showed that intradialytic exercise could increase 6MWD (MD = 36.65, 95%CI:24.90 to 48.39, *p* < 0.0001; Figure 5), with a low level of heterogeneity (I^2^ = 0%, *p* = 0.61). Egger’s test was non-significant (t = 0.62, *p* = 0.548). We also used subgroup analyses to assess the effects of different exercise types and showed that aerobic exercise, resistance exercise, and combined exercise all increased 6MWD, of these, studies of combined aerobic exercise and resistance exercise appeared to convey a higher improvement (MD = 57.21, 95%CI:26.68 to 87.73, *p* < 0.001; Figure 5).

#### 3.4.4 Physical activity

**FIGURE 6:**
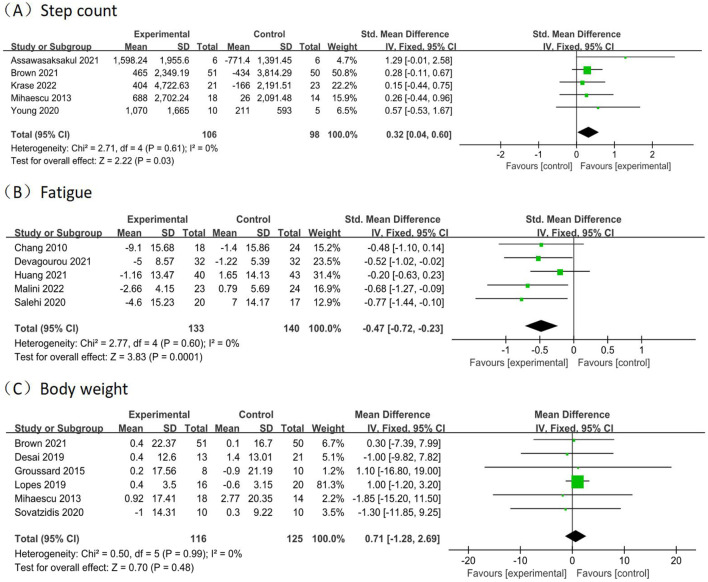
Forest plots of outcomes in step count, fatigue, and body weight. Forest plots of outcomes **(A)** step count; **(B)** fatigue; **(C)** body weight.

A total of 5 studies (Assawasaksakul et al., 2021; Brown et al., 2021; Krase et al., 2022; Mihaescu et al., 2013; Young et al., 2020) reported physical activity, as determined by step counts, involving 204 participants (106 in the experimental groups and 98 in the control groups). The analysis of data in the fixed effect model showed that intradialytic exercise could increase step counts (SMD = 0.32, 95%CI:0.04 to 0.60, *p* = 0.03; Figure 6A), with a low level of heterogeneity (I^2^ = 0%, *p* = 0.61). Egger’s test was non-significant (t = 1.86, *p* = 0.160).

#### 3.4.5 Fatigue

A total of 5 studies (Chang et al., 2010; Devagourou et al., 2021; Huang et al., 2021; Malini et al., 2022; Salehi et al., 2020) reported fatigue, involving 273 participants (133 in the experimental groups and 140 in the control groups). The analysis of data in the fixed effect model showed that intradialytic exercise could reduce fatigue (SMD = −0.47, 95%CI: 0.72 to −0.23, *p* = 0.0001; Figure 6B), with a low level of heterogeneity (I^2^ = 0%, *p* = 0.60). Egger’s test was non-significant (t = −3.00, *p* = 0.058).

#### 3.4.6 Body weight

A total of 6 studies (Brown et al., 2021; Desai et al., 2019; Groussard et al., 2015; Lopes et al., 2019; Mihaescu et al., 2013; Sovatzidis et al., 2020) reported body weight, involving 241 participants (116 in the experimental groups and 125 in the control groups). The analysis of data in the fixed effect model showed no significant effects in the body weight (MD = 0.71, 95%CI: 1.28 to 2.69, *p* = 0.48; Figure 6C), with a low level of heterogeneity (I^2^ = 0%, *p* = 0.99). Egger’s test was non-significant (t = −2.73, *p* = 0.053).

### 3.5 Publication bias

**FIGURE 7:**
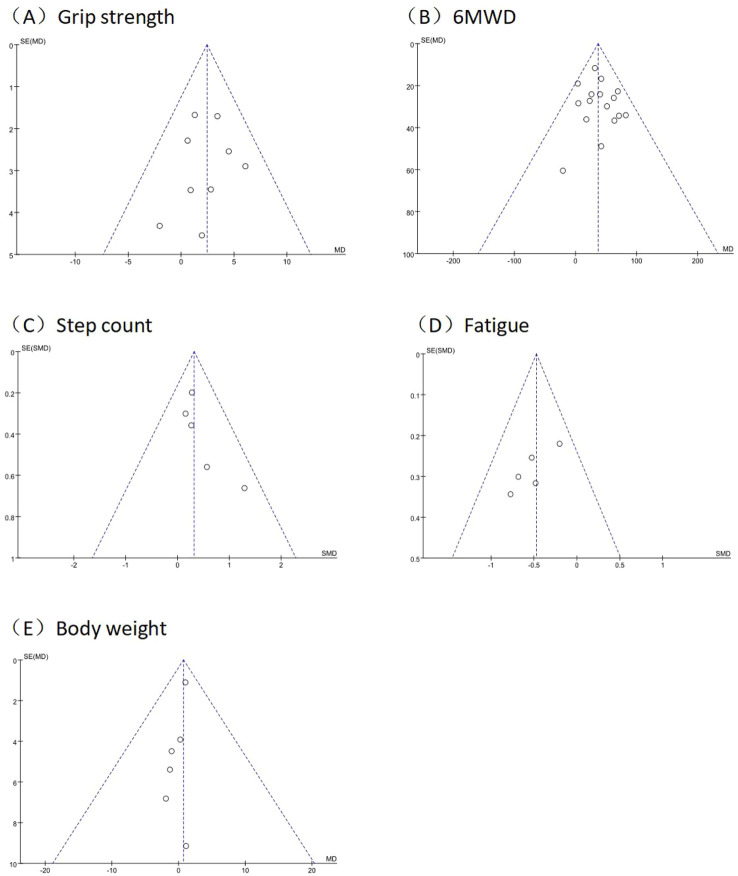
Funnel plot of outcomes in grip strength, 6MWD, step count, fatigue, and body weight. Funnel plots of **(A)** grip strength; **(B)** 6MWD; **(C)** step count; **(D)** fatigue; **(E)** body weight.

Funnel plots of grip strength, 6MWD, step count, fatigue, and body weight checked the possibility of publication bias, showed that the symmetry of the funnel plots is all better, and all Egger’s tests were non-significant (*p* > 0.05). suggests that there may be a low risk of publication bias. The results of funnel plots are detailed in Figure 7.

### 3.6 Sensitive analysis

Sensitivity analyses were conducted by sequentially excluding studies based on outcome indicators, and using the random-effects model or fixed-effects model to re-perform the analysis. The results showed that the statistical conclusions did not show any change. Hence, the meta-analysis results are stable.

## 4 Discussion

The prevalence of frailty is high in MHD patients, necessitating the establishment of systematic strategies for frailty identification and intervention. Exercise interventions can improve frailty by enhancing muscle strength and increasing physical activity levels. MHD patients receive an average of 12 h of hemodialysis treatment per week. Intradialysis exercise during this time is more adherence and safer when professionally guided and supervised by healthcare professionals. The United Kingdom Clinical Practice Guidelines for Hemodialysis (Ashby et al., 2019) recommend that “all MHD patients without contraindications in hemodialysis centers should engage in intradialytic exercise as a therapeutic approach to increase physical function and quality of life”. This systematic review and meta-analysis included 31 studies covering aerobic exercise, resistance exercise, and combined exercise, the results confirmed that intradialysis exercise can significantly improve frailty in MHD patients.

The overall frailty status in clinical practice is primarily assessed using standardized scales, and commonly used tools include Fried’s Frailty Phenotype, Frail Scale, Clinical Frailty Scale, and Edmonton Frailty Scale. Among them, Fried’s Frailty Phenotype has a precise objective index and high operability and is most commonly used in clinical practice. Our study found 3 studies reported that intradialytic exercise could reduce frailty score (MD = −0.98, 95%CI: 1.90 to −0.06, *p* = 0.04). In addition, this conclusion was further supported by a study (Yabe et al., 2022), whose data demonstrated that intradialytic exercise reduced the proportion of frailty patients. In clinical practice, we can develop stratified exercise programs based on the severity of frailty assessed through the frailty phenotype, enabling more personalized interventions. This approach aims to enhance patients’ physical activity capacity and quality of life while reducing healthcare utilization. However, there is still a lack of high-quality studies on improving overall frailty in MHD patients through intradialytic exercise. Large-sample, multicenter RCTs are needed to validate this conclusion in the future.

Grip strength is a simple, rapid, and noninvasive standardized indicator for assessing muscle strength and is an independent predictor of all-cause mortality in MHD patients (Vogt et al., 2016). Grip strength is primarily measured using a grip strength meter, which can reflect the patient’s weakness status. Our study found that intradialytic exercise could increase grip strength in MHD patients (MD = 2.42, 95%CI:0.78 to 4.06, *p* = 0.004), which is consistent with the results of the Meta-analysis by (Li et al., 2024). The reason may be that exercise can promote muscle protein anabolism, increase muscle fiber cross-sectional area and muscle volume, reduce fat accumulation, and then improve muscle mass and strength, thus increasing grip strength (Wang and Johansen, 2019). Subgroup analyses found that aerobic exercise, and resistance exercise all increased grip strength, patients may choose their preferred exercise type.

The 6MWD is a commonly used indicator to assess muscle function, which reflects exercise tolerance. Its test results are significantly and positively correlated with hemodialysis patients’ survival quality. Epidemiological data show that each 100-m increase in walking distance reduces the risk of all-cause mortality by 5.3% (Noguchi et al., 2022). Our study found that intradialytic exercise could increase the 6MWD in MHD patients (MD = 36.65, 95%CI:24.90 to 48.39, *p* < 0.0001), which is consistent with the results of the Meta-analysis by (Araujo et al., 2024). The reason may be that exercise induces endocrine changes in skeletal muscle, promotes arterial vasodilatation, stimulates oxygen utilization, and then improves muscle function, reduces muscle atrophy, and improves muscular coordination and endurance, thus increasing the 6MWD (Rhee et al., 2019; Rivera-Brown and Frontera, 2012). Subgroup analyses found that aerobic exercise, resistance exercise, and combined exercise all increased 6MWD, we can guide patients to choose the appropriate exercise type according to their actual situation in clinical practice.

Step counts are an objective indicator to assess physical activity levels, characterized by easy operation and accurate monitoring. Daily physical activity is monitored directly by wearing a pedometer. Our study found that intradialytic exercise could increase step counts in MHD patients (SMD = 0.32, 95%CI:0.04 to 0.60, *p* = 0.03), which is consistent with the results of the Meta-analysis by (Lock et al., 2021). The reason may be that exercise improves muscle strength and endurance, improves the patient’s exercise capacity, and reduces sedentary behavior, thus increasing step counts (Young et al., 2020).

Fatigue is a highly prevalent symptom in MHD patients, with a combined incidence of 61% (Dou et al., 2023). It is an independent predictor of cardiovascular events and all-cause mortality in MHD patients (Bossola et al., 2015). Our study found that intradialytic exercise could reduce fatigue in MHD patients (SMD = −0.47, 95%CI: 0.72 to −0.23, *p* = 0.0001), which is consistent with the results of the Meta-analysis by (Wahida and Rumahorbo, 2022). The reason may be that exercise improves muscle oxidative phosphorylation and muscle mitochondrial structure, promotes myocardin heavy chain synthesis, and then increases metabolism and improves systemic blood circulation, thus reducing fatigue (Bossola et al., 2015). The literature included in our study was assessed using the dedicated Fatigue Scale for MHD patients rather than alternative indicators such as conventional depression scales or vitality dimensions. Therefore, a limited number of studies that met the criteria were included.

Body weight is a commonly used indicator to assess health status, and MHD patients often experience weight loss in a chronically frailty state. Our study found no significant effect of intradialysis exercise on improving body weight in MHD patients (MD = 0.71, 95%CI: 1.28 to 2.69, *p* = 0.48). The reason may be that the exercise intervention duration included in the study was short and has not yet had a significant effect on body weight. In addition, body weight may be affected by a combination of dietary, metabolic, psychological, and disease factors. Longer-period, high-quality RCTs are needed to explore the potential effects of intradialysis exercise on body weight in MHD patients in the future.

Our study has several limitations. Firstly, given the specificity of the exercise intervention in the included studies, it was challenging to implement blinding of participants and researchers. Some studies did not elaborate on the methods used to generate the random sequence and conceal the allocation, which may cause selective bias. Secondly, this study included only published literature, did not search for grey literature, and excluded non-English literature, which may lead to incomplete inclusion. The literature included in this study varied due to differences in the type, frequency, intensity, and duration of the exercise, which may have affected the results of the pooled analyses. Lastly, most of the pooled outcomes in this study assessed indicators of physical frailty, excluding cognitive, psychological, or social domains. The study conclusions cannot fully replace overall frailty.

In conclusion, our study found that intradialytic exercise can significantly improve overall frailty and frailty indicators such as grip strength, 6MWD, step counts, and fatigue in MHD patients, which helps prevent and delay the progression of frailty. In clinical practice, we can tailor exercise programs according to different frailty problems of patients, thereby achieving personalized rehabilitation through improvement in corresponding frailty indicators. Therefore, all MHD patients without contraindications should engage in intradialytic exercise and develop personalized exercise programs according to the patient’s physical condition. More high-quality studies are needed in the future to assess the effect of intradialytic exercise on overall frailty and may further compare the effects of different types of intradialytic exercise on frailty in MHD patients.

## Data Availability

The original contributions presented in the study are included in the article/Supplementary Material, further inquiries can be directed to the corresponding author.
