# Supplementary material for: Effects of intradialytic exercise on frailty in maintenance hemodialysis patients: a systematic review and meta-analysis
Source: Front Physiol. 2025 Nov 6;16:1600219. doi: 10.3389/fphys.2025.1600219 (PMC12631337; doi:10.3389/fphys.2025.1600219)
Supplement: Supplementary file 1 [file Table1.docx]

Supplementary 1：Search strategy

1.PubMed

| Search ID | Search formula |
| --- | --- |
| #1 | (((((((exercise[MeSH Terms]) OR (training[Title/Abstract])) OR (intradialytic exercise[Title/Abstract])) OR (intradialytic training[Title/Abstract])) OR (aerobic exercise[Title/Abstract])) OR (aerobic training[Title/Abstract])) OR (resistance exercise[Title/Abstract])) OR (resistance training[Title/Abstract]) |
| #2 | (((((renal dialysis[MeSH Terms]) OR (hemodialysis[Title/Abstract])) OR (hemodialyses[Title/Abstract])) OR (haemodialysis[Title/Abstract])) OR (hemodiafiltration[Title/Abstract])) OR (haemodiafiltration[Title/Abstract]) |
| #3 | ((((frailty[MeSH Terms]) OR (frailties[Title/Abstract])) OR (frailness[Title/Abstract])) OR (debility[Title/Abstract])) OR (debilities[Title/Abstract]) |
| #4 | (hand strength[MeSH Terms]) OR (grip strength[Title/Abstract]) |
| #5 | ((walking speed[MeSH Terms]) OR (gait speed[Title/Abstract])) OR (walking pace[Title/Abstract]) |
| #6 | ((activities of daily living[MeSH Terms]) OR (daily living activity[Title/Abstract])) OR (physical activity[Title/Abstract]) |
| #7 | ((fatigue[MeSH Terms]) OR (lassitude[Title/Abstract])) OR (exhaustion[Title/Abstract]) |
| #8 | (body weight[MeSH Terms]) OR (body mass[Title/Abstract]) |
| #9 | #3 or #4 or #5 or #6 or #7 or #8 |
| #10 | #1 and #2 and #9  Filters:Publication Year from 2010 to 2024. |

2.Embase

| Search ID | Search formula |
| --- | --- |
| #1 | 'exercise'/exp OR ‘training’:ti,ab,kw OR ‘intradialytic exercise’:ti,ab,kw OR ‘intradialytic training’:ti,ab,kw OR ‘aerobic exercise’:ti,ab,kw OR ‘aerobic training’:ti,ab,kw OR ‘resistance exercise’:ti,ab,kw OR ‘resistance training’:ti,ab,kw |
| #2 | 'hemodialysis'/exp OR 'hemodialyses’:ti,ab,kw OR ‘haemodialysis’:ti,ab,kw OR ‘hemodiafiltration’:ti,ab,kw OR ‘haemodiafiltration’:ti,ab,kw |
| #3 | 'frailty'/exp OR ‘frailties’:ti,ab,kw OR ‘frailness’:ti,ab,kw OR ‘debility’:ti,ab,kw OR ‘debilities’:ti,ab,kw |
| #4 | 'hand strength'/exp OR ‘grip strength’:ti,ab,kw |
| #5 | 'walking speed'/exp OR ‘gait speed’:ti,ab,kw OR ‘walking pace’:ti,ab,kw |
| #6 | 'daily life activity'/exp OR ‘daily living activity’:ti,ab,kw OR ‘physical activity’:ti,ab,kw |
| #7 | 'fatigue'/exp OR ‘lassitude’:ti,ab,kw OR ‘exhaustion’:ti,ab,kw |
| #8 | 'body weight'/exp OR ‘body mass’:ti,ab,kw |
| #9 | #3 or #4 or #5 or #6 or #7 or #8 |
| #10 | #1 and #2 and #9 |
| #11 | #10 AND (2010:py OR 2011:py OR 2012:py OR 2013:py OR 2014:py OR 2015:py OR 2016:py OR 2017:py OR 2018:py OR 2019:py OR 2020:py OR 2021:py OR 2022:py OR 2023:py OR 2024:py) |

3.Web of science

| Search ID | Search formula |
| --- | --- |
| #1 | TS= (exercise OR training OR intradialytic exercise OR intradialytic training OR aerobic exercise OR aerobic training OR resistance exercise OR resistance training) |
| #2 | TS= (hemodialysis OR hemodialyses OR haemodialysis OR hemodiafiltration OR haemodiafiltration) |
| #3 | TS= (frailty OR frailties OR frailness OR debility OR debilities OR hand strength OR grip strength OR walking speed OR gait speed OR walking pace OR daily living activity OR physical activity OR fatigue OR lassitude OR exhaustion OR body weight OR body mass) |
| #4 | #1 and #2 and #3  Stipulated time: 2010-2024. |

4.Cochrane

| Search ID | Search formula |
| --- | --- |
| #1 | MeSH descriptor: [Exercise] explode all trees |
| #2 | (training):ti,ab,kw OR (intradialytic exercise):ti,ab,kw OR (intradialytic training):ti,ab,kw OR (aerobic exercise):ti,ab,kw OR (aerobic training):ti,ab,kw OR (resistance exercise):ti,ab,kw OR (resistance training):ti,ab,kw |
| #3 | #1 or #2 |
| #4 | MeSH descriptor: [Renal Dialysis] explode all trees |
| #5 | (hemodialysis):ti,ab,kw OR (hemodialyses):ti,ab,kw OR (haemodialysis):ti,ab,kw OR (hemodiafiltration):ti,ab,kw OR (haemodiafiltration):ti,ab,kw |
| #6 | #4 or #5 |
| #7 | MeSH descriptor: [Frailty] explode all trees |
| #8 | MeSH descriptor: [Hand Strength] explode all trees |
| #9 | MeSH descriptor: [Walking Speed] explode all trees |
| #10 | MeSH descriptor: [Activities of Daily Living] explode all trees |
| #11 | MeSH descriptor: [Fatigue] explode all trees |
| #12 | MeSH descriptor: [Body Weight] explode all trees |
| #13 | (frailties):ti,ab,kw OR (frailness):ti,ab,kw OR (debility):ti,ab,kw OR (debilities):ti,ab,kw OR (grip strength):ti,ab,kw OR (gait speed):ti,ab,kw OR (walking pace):ti,ab,kw OR (daily living activity):ti,ab,kw OR (physical activity):ti,ab,kw OR (lassitude):ti,ab,kw OR (exhaustion):ti,ab,kw OR (body mass):ti,ab,kw |
| #14 | #7 or #8 or #9 or #10 or #11 or #12 or #13 |
| #15 | #3 and #6 and #14  Filters:Publication Year from 2010 to 2024. |
